# Supplementary material for: Whole genome resequencing data enables a targeted SNP panel for conservation and aquaculture of Oreochromis cichlid fishes
Source: Aquaculture. 2022 Feb 15;548:737637. doi: 10.1016/j.aquaculture.2021.737637 (PMC8655616; doi:10.1016/j.aquaculture.2021.737637)
Supplement: Supplementary file 1 — Supplementary material 1 Table S1. Sample information, sequencing details and species assignments for each individual sampled for either panel design or panel testing according to morphological identification as well as the 96 SNP, 118 SNP, microsatellite and full-genome datasets. Table S2. Number of reads and mapping statistics for the whole genome sequence data. Table S3. Genome locations in both versions of the O. niloticus reference genome assembly (GCA_000188235.2 and GCF_001858045.2) and pairwise Fst values for the 120 SNPs. Table S4. Primer and probe sequences for all validated panels. Table S6. Individuals with differing assignments between fastSTRUCTURE and NewHybrids. P1 and P2 refer to the species an individual has ancestry components corresponding to in either the fastSTRUCTURE or NewHybrids analysis. Table S7. NewHybrids results for each individual in urolepis x leucostictus or urolepis x niloticus comparisons. [file mmc1.docx]

Figure S1. fastSTRUCTURE analysis and log-likelihoods for all individuals in the 96 SNP panel dataset, from *K*=2 to *K*=5.

*.*

Figure S2. ΔK (DeltaK) values for STRUCTURE runs on the microsatellite dataset, without prior assignment, from *K*=2 to *K*=6.

Figure S3. Comparison between species assignment using the 96 SNP panel (top row), and microsatellite analysis without prior assignment at *K*=3 (second row) & *K*=4 (third row). The fourth row gives a block colour for species assignment in each of the analysis: grey corresponds to hybrid, blue to *O. leucostictus*, red to *O.* *urolepis* and cyan to *O. niloticus*.

Figure S4. a) Average log-likelihoods for the 100 replicates of each number of sub-sample SNPs. Error bars represent standard error. b) The percentage of replicates for each number of sub-sample where all of the reference individuals were correctly assigned to their species.

Figure S5. a) The mean number of hybrids called (no ancestry component > 80%) across the 100 replicates of each number of sub-sample SNPs. Error bars represent standard error. b) The mean variability in minor ancestry component between the 100 replicates for each individual identified as hybrid in at least one of these replicates for each number of sub-sample SNPs. Error bars represent standard error. c) Histogram of the frequency at which hybrids were classified as hybrids across the 100 replicates of 96 random SNPs. d) same as c) except that data for individuals never identified as hybrids is added.
